# Academic dishonesty among academics in Malaysia: a comparison between healthcare and non-healthcare academics

**DOI:** 10.1186/s12909-018-1274-3

**Published:** 2018-07-17

**Authors:** John Jeh Lung Tiong, Hui Ling Kho, Chun-Wai Mai, Hui Ling Lau, Syed Shahzad Hasan

**Affiliations:** 1 School of Pharmacy, Taylor’s University, No. 1, Jalan Taylor’s, 47500 Subang Jaya, Selangor Malaysia; 2 School of Pharmacy, International Medical University, 126, Jalan Jalil Perkasa 19, Bukit Jalil, Kuala Lumpur Malaysia

**Keywords:** Academic dishonesty, Academics, Healthcare, Non-healthcare

## Abstract

**Background:**

This study was carried out to gauge the prevalence of academic dishonesty among academics in Malaysian universities. A direct comparison was made between academics of healthcare and non-healthcare courses to note the difference in the level of academic integrity between the two groups. In addition, the predisposing factors and implications of academic dishonesty, as well as the different measures perceived to be effective at curbing this problem were also investigated.

**Methods:**

A cross-sectional study design with mixed qualitative and quantitative approaches was employed and data collection was carried out primarily using self-administered questionnaire.

**Results:**

Approximately half (52.5%, *n* = 74) of all respondents (*n* = 141) reported having personally encountered at least one case of academic dishonesty involving their peers. The results also revealed the significantly higher prevalence of various forms of academic misconduct among healthcare academics compared to their non-healthcare counterparts. Although respondents were generally conscious of the negative implications associated with academic dishonesty, more than half of all cases of misconduct were not reported due to the indifferent attitude among academics. Low levels of self-discipline and integrity were found to be the major factors leading to academic misdeeds and respondents opined that university managements should be more proactive in addressing this issue.

**Conclusions:**

The outcome of this study should serve as a clarion call for all relevant stakeholders to start making immediate amends in order to improve the current state of affairs in academia.

## Background

Academic integrity is the fundamental commitment that should be upheld by all academics in their meticulous bid to instil professional integrity among future workforce [1]. The perception of professionalism among students is heavily influenced by the ethos of a university and its faculties [2]. In essence, students’ observations of the working etiquettes displayed by the academic fraternity will indirectly shape their professional identities [3–7]. Therefore, as the custodians of professional virtues and role models for students, the professional behaviour of academics should reflect the core values underlying the principles of academic integrity which encompass honesty, trust, fairness, respect and responsibility as defined by The Centre of Academic Integrity [8, 9]. Although it is imperative for academics to self-regulate, it remains challenging to measure the actual level of compliance with the code of ethics stipulated by the respective institutions [9, 10]. Nevertheless, media outlets in Malaysia have recently highlighted numerous dishonest acts among academics in both public and private universities in the country. The common cases reported include altering of assessment mark after taking a bribe, tampering with academic appraisal surveys, academic plagiarism, publication fraud and unethical authorship exchange [11–13]. Despite being based on circumstantial evidence, these undoubtedly cast a bad light on the image of academics whilst placing the country’s institutions of higher education in disrepute. It is worth noting that, the lack of official statistics pertaining to this matter is attributable to under-reporting by universities, as these issues tend to remains closeted. This is further compounded by the limited number of research on this topic despite being a common perception within the academic fraternity.

This study was carried out through a peer-reporting survey to gauge the prevalence of academic dishonesty among academics in Malaysian universities. The public generally holds high regards toward the healthcare professions according to the annual Gallup honesty/ethics poll [14, 15]. This is unsurprising, given the well-established governance structure regulating both the profession and the professional education [16, 17]. Professional integrity among healthcare workers is mandatory and non-conceding considering the nature of the job. Thus, the level of integrity expected of healthcare academics who act as role models for the future healthcare workforce, should not be any lesser. While there have been on-off reports of academic dishonesty among academics, there were no previous studies comparing between academics of healthcare and non-healthcare courses. If proven to be true, this will likely undermine the quality of professional training for future workforce regardless of a well-planned professional curriculum [18–20]. What more if it involves future healthcare professionals where any lapse in professionalism can detrimentally affect not only patient safety but also clinical outcomes [21, 22]. In addition, factors predisposing to dishonest acts as well as the different measures perceived to be effective in curbing this problem were also investigated. The outcome of this study may serve as an important feedback to the academic fraternity and all other relevant stakeholders for the continuous improvement of education quality in order to attain true world-class standards.

## Methods

### Study design

A cross-sectional design with mixed qualitative and quantitative approach was used to explore the prevalence of academic dishonesty among academics from numerous universities in Malaysia. In order to gain an insight into the climate of academic integrity within the academic fraternity in Malaysia, academics from both healthcare (Medicine, Pharmacy and Dentistry) and non-healthcare courses from six universities were recruited in this study.

### Study instrument

The study questionnaire was designed with reference to a previous study published by Archibong and co-workers which consisted of both closed-ended and open-ended questions [23]. The questionnaire has been modified to meet the current study objectives. First section of the questionnaire contained questions for extraction of (1) demographic information of participants (gender, age, years of teaching experience, academic rank and faculty). The subsequent four sections comprise of questions that aimed to evaluate (2) the prevalence of academic dishonesty among lecturers and reporting rate, (3) its perceived adverse implications, and (4) its predisposing factors as well as measures proposed to tackle the issue. Likert scale was utilized in the quantitative analysis in the five different sections whereas open-ended questions were included to elicit contextual information regarding respondents’ opinions in various aspects of the study.

### Scoring of instrument’s items

Information pertaining to the prevalence of academic dishonesty was sought through asking respondents if they have ever heard or personally encountered any form of dishonest acts among their peers. Each form of academic misconduct was scored from 0 to 1, where 0 = have not heard/encountered; 0.5 = hearsay/rumours; 1 = direct personal encounter. The respondents were also asked to rate the perceived likelihood that academic dishonesty among academics might adversely affect the university. This was then scored from 0 to 3 where, 0 = Does not affect at all; 1 = Mildly affect; 2 = Moderately affect; 3 = Severely affect. The significance of different factors predisposing to the prevalence of academic misconduct among academics were rated by respondents and subsequently scored from 1 to 3 where, 1 = Low significance; 2 = Moderate significance; 3 = High significance.

### Validity and reliability

A total of 14 academics were recruited in a pilot study for questionnaire content validation and reliability testing. This was done to ensure that the questions were clear, easy to comprehend and sufficient to meet the study objectives. In addition, the questionnaire was also face validated and analyzed by two independent researchers. Reliability test was conducted using test-retest reliability method. Cohen’s kappa coefficient was used to determine the reliability of the questionnaire using SPSS and it yielded a value of 0.679 which reflected a substantial agreement of reliability [24].

### Data collection process

During the data collection phase, one of the researchers approached each participating university to gather the required information. The study questionnaire was sent to the coordinators at six participating universities, with a copy of the ethical approval letter, participant information sheets and consent forms. Convenience sampling was used to enrol all the eligible respondents during the study period. Due to the sensitive nature of this study, names of the participating institutions cannot be disclosed. Only the participants who have signed the ‘written consent form’ with a completed questionnaire were included in this study. Respondents were assured of their anonymity and data confidentiality to elicit honest response. They were informed that their participation was voluntary and completion of the questionnaire was optional to protect the respondents’ right to privacy. To further ensure confidentiality, only selected researchers in the team have access to the collected data.

### Statistical analysis

Both descriptive and inferential data analyses were carried out using Statistical Package for Social Sciences, SPSS® version 18 with 0.05 as the level of significance. Descriptive statistics were used to analyse frequency, percentage, mean, percentile, range and standard deviation. Independent student *t*-test and/or one-way analysis of variance (ANOVA), followed by post hoc *analyses* using Least Statistical Difference (LSD).

## Results

### Demographics of study population

Of over 1000 staff approached, only 141 academics from six Malaysian universities accepted and completed the study questionnaire, with an overall response rate of 13.5%. The demographics of respondents are summarized in Table 1. Among those who participated, 60.2% had teaching experience of 9 years or lesser and were mainly at the ranks of lecturers or senior lecturers (95.1%).

**Table 1:** Demographics of respondents

	Healthcare Academic n (%)	Non-healthcare Academic n (%)
Total	68 (48.2%)	73 (51.8%)
Male	23 (16.3%)	30 (21.3%)
Female	45 (27.9%)	43 (30.5%)
Years of Teaching Experience		
< 5 years	25 (17.7%)	11 (7.8%)
5–9 years	24 (17.0%)	25 (17.7%)
10–14 years	9 (6.4%)	13 (9.2%)
15–19 years	4 (2.8%)	9 (6.4%)
> 19 years	6 (4.3%)	15 (10.6%)
Academic Ranking		
Lecturer	36 (25.5%)	28 (19.9%)
Senior Lecturer	25 (17.7%)	40 (28.4%)
Associate Professor	5 (3.5%)	2 (1.4%)
Professor	2 (1.4%)	3 (2.1%)

### The prevalence of academic dishonesty and reporting rate among academics

#### i) Prevalence of dishonest acts

**Fig. 1:**
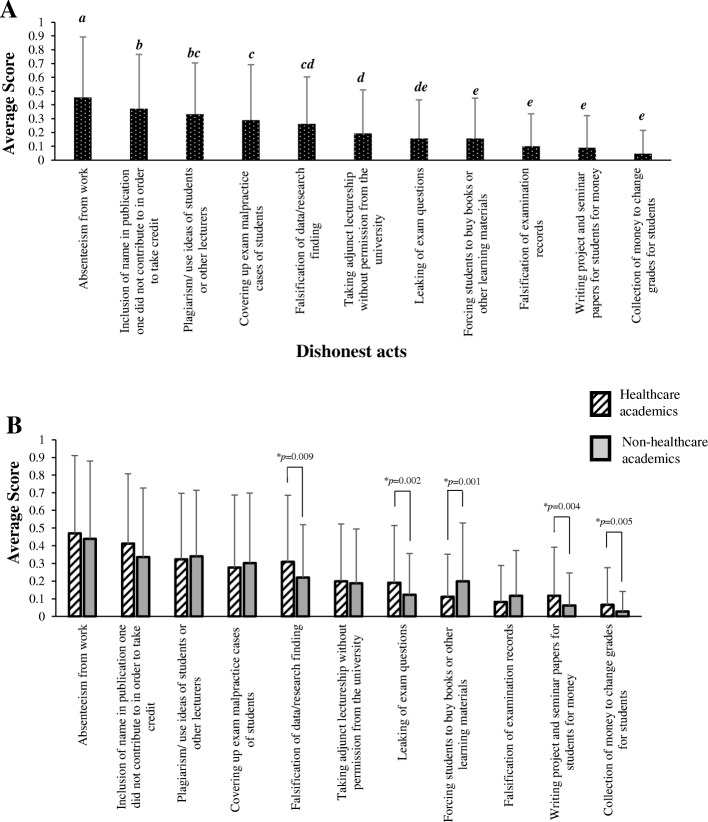
Prevalence of dishonest acts among academics. **a** The prevalence of any form of dishonest acts either encountered personally or through hearsay among peers, as reported by respondents (*n* = 141). Each form of academic misconduct was scored from 0 to 1, where 0 = have not heard/encountered; 0.5 = Hearsay/rumours; 1 = Direct personal encounter. Bars represent the average score ± standard deviation for each dishonest act. The dishonest acts were ranked from the most encountered (highest score) to the least encountered (lowest score). Different lowercase letters above the error bars indicate statistically significant differences (*p* < 0.05; Least Statistical Difference (LSD) post hoc test). **b** The average score of each dishonest act ± standard deviation from healthcare academics was compared to non-healthcare academics. Asterisks (*) indicate statistically significant differences (*p* < 0.05, independent t-test)

The scores for all forms of academic misconducts reported by respondents are summarized in Fig. 1a. This study revealed that approximately half (52.5%) (*n* = 74, data not shown) of all respondents conceded to having personally encountered at least one case of academic misconduct throughout their career. The data showed that the most common form of impropriety among academics is *absenteeism from work* (average score = 0.45) with its prevalence being significantly higher than all other forms of misconducts (*p* < 0.005). Other incidences of misconduct identified include *giving of publication authorship to non-contributor* (average score = 0.37), *academic plagiarism* (average score = 0.33), *covering up of student’s exam malpractice* (score = 0.29), *falsification of research data/finding* (average score = 0.26), *taking adjunct lectureship without permission from the university* (average score = 0.19), *leaking of exam questions* (average score = 0.16), *forcing students to buy books or other learning materials* (average score = 0.16), *falsifying exam records* (average score = 0.1), *writing student assignments for money* (average score = 0.09) and *accepting bribes to change student grades* (average score = 0.05).

The study also revealed the significantly higher prevalence of academic dishonesty among healthcare academics compared to their non-healthcare peers in the forms of *falsification of research data/finding* (*p* = 0.009), *leaking of exam questions* (*p* = 0.002), *writing student assignments for money* (*p* = 0.004) *and accepting bribes to change student grades* (*p* = 0.005) (Fig. 1b). Meanwhile, there was a higher prevalence of misconduct in terms of *forcing students to buy books or other teaching materials* (*p* = 0.001) for monetary gain among non-healthcare academics compared to healthcare academics.

All other forms of academic dishonesty reported by healthcare academics and non-healthcare academics identified through the open-ended question were grouped according to common themes (Table 2).

**Table 2:** Thematic analysis of other forms of academic dishonesty among academics based on respondents’ feedback

Theme	Comments
Giving of unjust marks to students	“*Giving more marks than deserved*.” (HC, Senior Lecturer) “*Did not carefully grade student’s work*.” (NH, Senior Lecturer)
Bending rules for some students/practising favouritism toward certain students	“*Bending the rules for some students, such as allowing students to submit assignments months later without penalty, in order to ensure that the whole class loves the lecturer and gives the lecturer a good assessment [evaluation].*” (HC, Lecturer) “*Lecturers practicing favouritism among students. Favourite students tends [tend] to get more exam info/hints, more attention in class and after class compared to other students, not penalised*” (HC, Senior Lecturer)
Using research grants for personal purposes	“*Use research money for personal purposes*.” (NH, Senior Lecturer)
Covering up misconducts of colleagues	“*Cover up for friends when they did wrong*” (HC, Associate Professor)

*Abbreviations: HC* healthcare academic, *NH* non-healthcare academic

#### ii) Academic dishonesty reporting rate

This study also uncovered that 44.1 and 43.8% of all cases of misconduct encountered by healthcare and non-healthcare academics respectively, were not reported to the authority/management. The views of some of the participants which explains the factors influencing the decision in reporting academic dishonesty among peers:

“*The boss always have [has] better say and approach to higher authority than juniors. As a result of which no juniors dare to complain. They just mold themselves and [are] bound to do unethical things*.”

“*Nobody wants to be a whistle-blower, and the Asian culture makes it not socially acceptable to ruin a person’s ‘rice bowl’ [career] with reporting of an untoward incident*.”

“*Its[it’s] none of my business*”

*“Assumption that [it] is a normal and acceptable practice”*

Other reasons for the lack of misconduct reporting cited by the respondents through the open-ended question were grouped according to common themes (Table 3).

**Table 3:** Thematic analysis of the reasons for the low reporting rate of academic dishonesty based on respondents’ feedback

Theme	Comments
Covered up to protect the name of the institution	“*To preserve the good name of the school/university*” (HC, Senior Lecturer)
Difficulty in establishing evidence	*“No hard evidence, or only hearsay, sabotage by fellow colleagues.”* (HC, Senior Lecturer)
Weakness in the reporting system	“*No proper channel to report such things. I inquire to HR before, they said will get back to me but never did*.” (NH, Senior Lecturer) “*Lazy to do all the paperwork and too time consuming*” (NH, Senior Lecturer)
No action expected to be taken against the perpetrator	*“Nothing is going to be done about it.”* (HC, Associate Professor)
The belief of giving others a second chance	*“To give opportunity for change due to other person [*sic*] mistakes”* (NH, Senior Lecturer)

*Abbreviations: HC*, healthcare academic, *NH* non-healthcare academic

### Adverse implications caused by dishonesty among academics

**Fig. 2:**
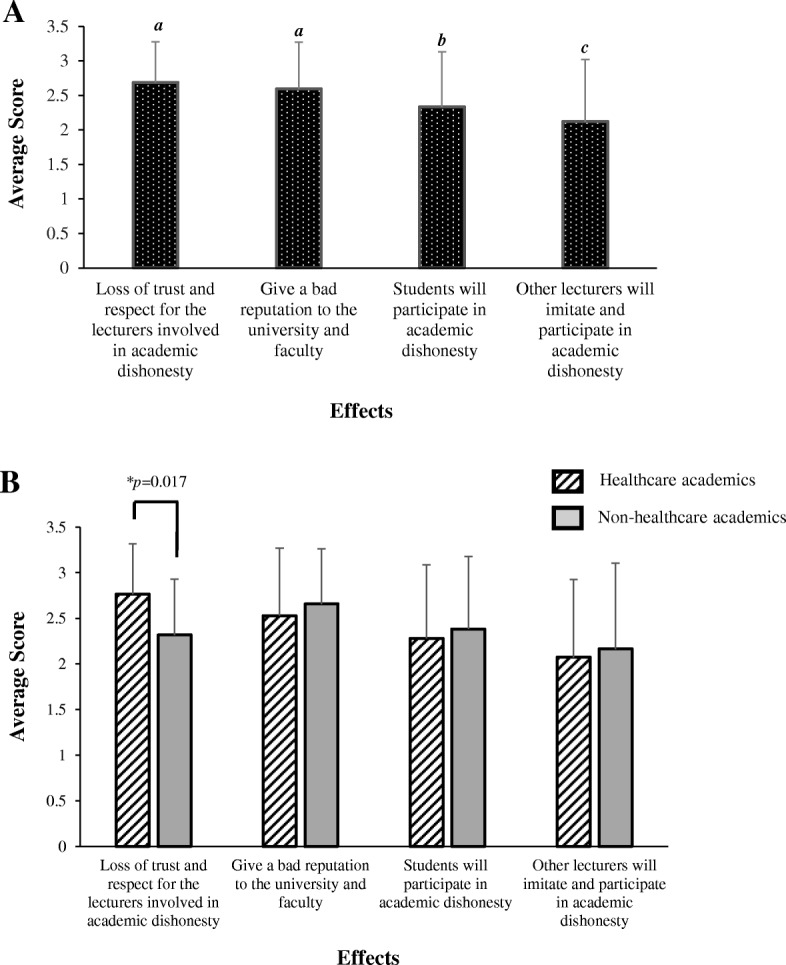
The adverse implications caused by academic dishonesty among academics. **a** The perceived severity of an adverse effect affecting the university as a result of academic dishonesty, as rated by respondents (*n* = 141). This was scored from 0 to 3 where, 0 = Does not affect at all; 1 = Mildly affect; 2 = Moderately affect; 3 = Severely affect. Bars represent the average score ± standard deviation of each effect. The effects were ranked from the most likely (highest score) to the least likely (lowest score). Different lowercase letters above the error bars indicate statistically significant differences (*p < 0.05; Least Statistical Difference (LSD) post hoc test). **b** The average score of each perceived effect ± standard deviation from healthcare academics was compared to non-healthcare academics. Asterisks (*) indicate statistically significant differences (*p* < 0.05, independent t-test)

The most severe adverse implication was thought to be *the loss of trust and respect for the lecturers involved in academic dishonesty* (average score = 2.69) (Fig. 2a). It is worth noting that healthcare academics believed in a more deleterious effect in this aspect compared to their non-healthcare counterparts (*p* = 0.017) (as shown in Fig. 2b). Likewise, the respondents also believed that the *reputation to the university and faculty will be affected* (average score = 2.60), followed by *spurring students’ participation in academic misconduct* (average score = 2.33) and *spurring other lecturers to initiate and participate in academic misconduct* (average score = 2.12).

### Factors predisposing to academic dishonesty and the proposed measures to tackle this issue

#### i) Factors predisposing to the dishonest acts among academics

**Fig. 3:**
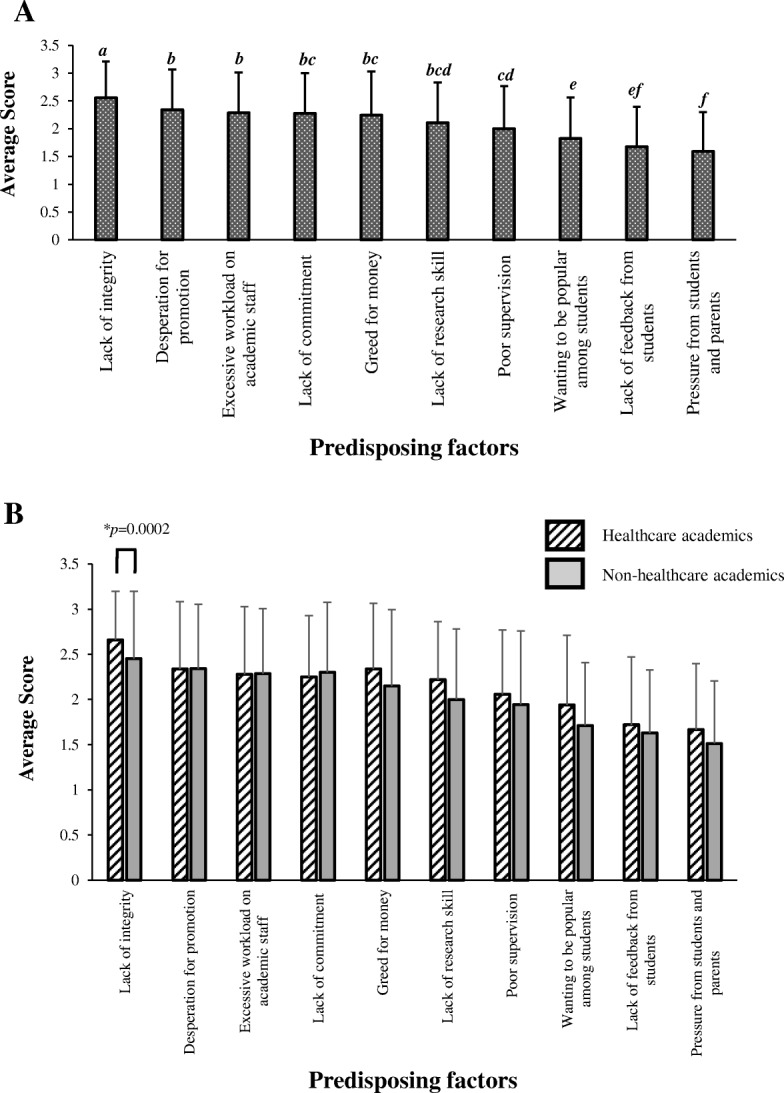
The factors predisposing to the prevalence of academic misconduct among academics. **a** The perceived significance of various predisposing factors leading to academic misconduct among academics, as rated by respondents (*n* = 141). This was scored from 1 to 3 where, 1 = Low significance; 2 = Moderate significance; 3 = High significance. The factors were ranked from the most likely factor (highest score) to the least likely factor (lowest score). Different lowercase letters above the error bars indicate statistically significant differences (*p < 0.05; Least Statistical Difference (LSD) post hoc test). **b** The average score of each predisposing factor ± standard deviation from healthcare academics was compared to non-healthcare academics. Asterisks (*) indicate statistically significant differences (*p* < 0.05, independent t-test)

The most significant factor (Fig. 3a) was perceived to be the *lack of discipline/moral/ integrity* (average score = 2.55), followed by *desperation for promotion* (average score = 2.34), *excessive workload* (average score = 2.28), *lack of commitment* (average score = 2.28), *greed for money* (average score = 2.24), *lack of research skill* (average score = 2.11), *poor supervision by superior* (average score = 2.00), *wanting to be popular among students* (average score = 1.82), *lack of feedback from students* (average score = 1.67) and *pressure from students and parents* (average score = 1.59). Figure 3b showed that healthcare academics viewed *lack of discipline/moral/ integrity* as a more significant factor that predisposes to academic dishonesty compared to their non-healthcare counterparts (*p* = 0.0002). The perception towards other predisposing factors was comparable between both healthcare and non-healthcare academics.

Other factors contributing to the propensity of academic dishonesty as opined by several respondents (in the open-ended question and grouped according to common theme) were included in Table 4. The findings unveiled low levels of academic integrity and/or the poor understanding of its principle among academic staff. The views of several respondents echoing such sentiment were highlighted below:

**Table 4:** Thematic analysis of other factors predisposing to the dishonest acts among academics based on respondents’ feedback

Theme	Comments
Lack of awareness/ understanding of what constitutes academic dishonesty	“*Lecturers from different cultures may have different interpretation[s] of academic dishonesty*.” (HC, Lecturer) “*Lack of awareness of their actions are considered academic dishonesty*” (HC, Lecturer)
Empathise with low grade students	“*Wish to help a friend/low grade student*” (NH, Senior Lecturer)
Dishonest/corruption at higher level	“*when dishonesty and corruption is carried out in [happen at] the highest level....you can’t change much*” (HC, Senior Lecturer)
Stressful working condition	*“High[ly] demanding key performance index for lecturers which might force the lecturers to conduct (*sic*) dishonesty*.” (HC, Senior Lecturer)
Lack of recognition and appreciation of staff contribution	“*Lack of recognition and appreciation for the staff will lead to dishonesty especially for the senior lecturers*” (NH, Lecturer)

*Abbreviations: HC*, healthcare academic, *NH* non-healthcare academic

*“In the absence of specific code/guidelines, lecturers may undertake activities that compromise good governance. It is also useful to know what the university’s philosophy is especially in areas where there are much (sic) ambiguities”*

*“The code of ethics will serve as standards to foster mutual trust, to encourage free exchange of ideas as well as to advance the quest for knowledge by requiring intellectual honesty in teaching, learning and research”*

#### ii) Measures proposed by respondent to tackle academic dishonesty among academics

The various measures proposed by the respondents through the open-ended question were summarized according to common themes in Table 5.

**Table 5:** Thematic analysis of measures perceived to be effective for tackling academic dishonesty based on respondents’ feedback

Theme	Comments
Proper implementation/enforcement of rules and policy	*“Code of ethics and standards are only written on handbook; however, the enforcement of the code of ethics and standard are (has) not materialized and (has) not (been) implemented”* (NH, Lecturer) *“Having a policy cannot be used as a guarantee that lecturers will always uphold their integrity. It is only to what extent that the policy is been practiced that counts”* (HC, Lecturer) *“There are rules and regulations but across the board, enforcement is an issue”* (HC, Professor) “*Thorough investigation and immediate action*” (NH, Professor) “*Protect the whistle blower; Reward the honesty such as being honest (*sic*)*.” (NH, Senior Lecturer)
Proper channels/procedures to report/investigate	“*More avenues for anonymous feedback from other academics and students on academic dishonesty*” (NH, Senior Lecturer)
Peer review	“*Peer review system*” (HC, Senior Lecturer)
Higher authority/management should lead by example	“*Good examples of academic integrity from senior lecturers can be the best guidance to all fresh lecturers.”* (NH, Senior Lecturer) “*The management team of the university must commit themselves towards academic integrity of the academic staff - take action when required*” (NH, Senior Lecturer)
Rotation of leadership positions to prevent complacency	“*Rotate the headship to prevent complacency*” (HC, Senior Lecture)

*Abbreviations: HC*, healthcare academic, *NH* non-healthcare academics

## Discussion

### The prevalence of academic dishonesty and reporting rate among academics

#### i) Prevalence of dishonest acts

The findings of this study corroborated the high prevalence of academic dishonesty among academics in universities previously reported by several media outlets [11, 12]. This appears to concur with a meta-analysis which also reported the higher frequency of misconducts among medical/pharmacological researchers compared to their peers in other fields [25]. This is alarming considering that educators are monetizing their influence on selection of teaching materials when priority for such selection should be based on their suitability for optimal student learning experience. With academic integrity of academics being the cornerstone in fostering the same level of professional behaviour among students, the lack of such attributes, especially within the healthcare academic fraternity could result in a foreseeable lapse of professionalism among future healthcare professionals. This could be further exacerbated by the supervision-deprived professional training among students caused by the absenteeism among academics as reported above.

The examples of other misconducts named by participants were often associated with compromised teaching quality such as unprofessional grading or giving undeserving marks to students; bending of rules for selected students or favouritism; misuse of research grant funding and; covering up of another academics’ misconduct. These behaviours were seen to be driven by the desire to gain favour from students, possibly in attempts to obtain a good assessment score as part of the academic’s performance evaluation.

#### ii) Academic dishonesty reporting rate

Despite being common knowledge, academic dishonesty is often not openly discussed/reported probably due to its sensitive nature [9]. The low reporting rate is another testament of the sensitivity and controversy surrounding such matters, which can be complicated by the fear of reprisals towards whistle-blowers or risk of creating unpleasant tension in their workplace. Moreover, responses from the participants indicated that there is an unhealthy perception of dishonesty being the ‘norm’; as well as the non-interference or ‘go-with-the-flow’ attitude within the academic community which could have resulted in higher tolerance towards acts of academic dishonesty. It was apparent from this study that academics generally feel compelled to safeguard both their own interest and that of their university. It is also a common belief that perpetrators are less likely to be held accountable for their misconducts owing to the lack of transparency in handling these cases, which could demotivate academics from reporting dishonest acts [9]. Meanwhile, the absence of proper channels and procedures for reporting, or unawareness towards these channels was seen as a further deterrent in reporting of academic dishonesty.

### Adverse implications caused by dishonesty among academics

Suffice to say, academics are well aware that their misdeeds cast a bad light not only on their own image and credibility as educators but also that of the universities; in addition to propagating the unwholesome culture of cheating in universities. The trust and respect vested on academics and graduates of these universities are also indirectly at stake [26]. The awareness of the associated ramifications among academics in addition to the reasons for not reporting academic dishonesty appear to suggest that both actions (flouting of ethical codes of and the lack of misconduct reporting) could be a conscious choice.

### Factors predisposing to academic dishonesty and the proposed measures to tackle this issue

#### i) Factors predisposing to the dishonest acts among academics

The findings unveiled low levels of academic integrity and/or the poor understanding of its principle among academic staff which is believed to have led to flawed ethical judgment among academics resulting in injudicious decisions at workplace. The higher awareness among healthcare academics that low levels of discipline/moral/integrity is the primary factor predisposing to dishonest acts appears paradoxical, given the significantly higher prevalence of numerous types of transgression reported among them compared to non-healthcare academics (Fig. 1b). This finding highlights the general misconception that academics would by default behave professionally given their professional background and personal cognizance of their moral/ethical obligations towards the teaching profession [9]. This underscores the need of university to be committed to administering the fundamental values underlying academic integrity through effective staff orientation and training programmes emphasizing on the code of ethics whilst resetting the moral compass of problematic staff.

More importantly, the general disregard for ethical obligations among academics was found to be driven-primarily by self-interest which calls for a more humanistic approach to address the possible root causes rather than deterrent policies in addressing such problems.

The common cases of unscrupulous authorship exchange, academic plagiarism and falsification of research data on the other hand, also highlighted the dreadful ‘publish or perish’ phenomenon plaguing academia in the rat race for university global rankings [9, 20, 27]. Unknowingly, the over-emphasis of research output by the universities could have resulted in more time and effort being diverted from the teaching domain with the caveat of spurring potential misdeeds not only in attempts to conceal compromised teaching quality, but also in glamorising one’s research profile [9]. Unrealistic research-based key performance indices (KPI) within the academic fraternity have somewhat confirmed the long-surmised mismatched between the expectations of the academics and the cultures of universities. As such, universities should consider to realign the goals of both university and its academic staff by setting realistic KPI to prevent work stress-related misdeeds among teaching staff.

#### ii) Various measures proposed by respondent to tackle academic dishonesty among academics

The suggestions provided by participants pointed towards the general sentiments that a more proactive role by the university management in administering university policies would aid in tackling academic dishonesty [28]. This alludes to the fact that the mere existence of policies without proper implementation by institutional committee(s) is inadequate in curbing academic menace which concurs with the findings of previous research [21, 29]. This is also an indirect reflection of the widespread frustration among academics caused by the university’s apathy in dealing with such matters.

In this context, proper reporting channels should be made available to promote judicious reporting while investigation procedure and its outcome must be made transparent to restore the confidence of academics toward the system. There is also a dire need for impartial enforcement of policy whilst conferring protection upon whistle-blowers particularly if the perpetrator is a person of authority within the organization. More pertinently, only impeccably credentialed individuals with admirable integrity should hold leadership positions, making leadership by example a reality. The following is a summary of recommendations based on the various measures proposed by respondents:

- Provide proper channel and procedures for anonymous reporting of academic dishonesty.
- Any enquiry towards alleged academic dishonesty should be transparent and the outcome made known to preserve institution’s integrity.
- Setting of realistic goals for career progression for academics to prevent resorting to unscrupulous means to achieve these goals.
- Transparent and strict enforcement of the code of ethics by an independent body – punishment should not be dependent on one’s rank.
- Only individuals with the right credentials and professional integrity should be appointed to leadership positions in order to lead by example.

### Limitations

This research is the first of its kind, designed with the aim to compare the prevalence of academic dishonesty between academics of both healthcare and non-healthcare courses. Moreover, the use of mixed quantitative and qualitative approaches in this peer-reporting survey had elicited a more comprehensive ‘on-the-ground’ insight into the predisposing factors, implications as well as numerous proposed measures for tackling such issue. The sentiments shared by respondents would therefore be useful for universities in devising more effective and targeted interventions. It is worth noting that the data of this study may represent a lower estimate of the true prevalence given the lower-than-expected participation rates due to the sensitive questions being asked in the survey. Undoubtedly, a larger sample size with sampling centres across the country would provide a better reflection of academic dishonesty in Malaysia.

It would be interesting for future studies to address the following issues which would no doubt provide a clearer insight for devising remedial actions to counter academic dishonesty:

- The psychological barriers among academics which post hindrance to their voluntary participation in order to secure a higher response rate.
- The reasons underlying the higher prevalence of dishonesty among academics teaching healthcare courses compared to their counterparts in non-healthcare courses.
- The opinion among academics on what constitutes as practical procedures or relevant outlets for confidential reporting of academic misdeeds encountered.
- The perception among management of universities and academics regarding severity of misconducts among academics and perceived justifiable penalty towards those with reprimandable behavior.

## Conclusions

The outcome of this research has reaffirmed the open secret pertaining to the dishonest acts among academics with the irony of them supposedly being role models for character and intellectual development among university students. Of greater concern is the overall higher prevalence of lapses in academic integrity among healthcare academics despite their highly esteemed professional background. In view of this, all institutions of higher learning should embody a clear definition of academic integrity in the university tenets and to ensure that its underlying core values are upheld at all times. Nevertheless, university policies must also be viewed as egalitarian and practical in order to foster compliance. Reference should be made to outcomes of existing studies and other exemplary guidelines in the drafting of institutional policies that can be relied on to promote transparency in addition to averting potential conflicts of interest in the academic fraternity. More importantly, university management should take heed of the feedback given by the academics such as those elicited by this study by taking immediate actions to address the issues.

## Abbreviations

ANOVA: Analysis of variance
HC: Healthcare academic
LSD: Least Statistical Difference
NH: Non-healthcare academic
